# Editorial: The Patient's Change: Understanding the Complexity of the Dynamics of Change and Its Precursors in Psychotherapy

**DOI:** 10.3389/fpsyg.2021.739727

**Published:** 2021-09-13

**Authors:** Giulio de Felice, Melissa M. De Smet, Reitske Meganck, Guenter Schiepek

**Affiliations:** ^1^Department of Dynamic and Clinical Psychology, Sapienza University of Rome, Rome, Italy; ^2^Faculty of Psychology, NC IUL London, London, United Kingdom; ^3^Department of Psychoanalysis and Clinical Consulting, Ghent University, Ghent, Belgium; ^4^Institute of Synergetics and Psychotherapy Research, Paracelsus Medical University, Salzburg, Austria; ^5^Department of Psychology, Ludwig Maximilian University of Munich, Munich, Germany

**Keywords:** change in psychotherapy, precursors of change, dynamics of change, psychotherapy process, psychotherapy process-outcome research, dynamic systems

It is really a great pleasure for us to introduce this Research Topic focused on the dynamics of change in psychotherapy. This Research Topic hosts contributions by authors from different parts of the world. We thank them for giving us the opportunity to learn from their latest research and clinical reflections. The specific theme of this Research Topic is a pivotal and pressing question in psychotherapy research: What are the processes that promote change in psychotherapy and what are their precursors? The scientific literature, up to now, has no coherent and univocal answer to this matter. Therefore, this Research Topic presents systematic and wide-ranged analyzes related to these questions. In this editorial, we extract some common characteristics present in the empirical and clinical research of the contributors, with the aim of providing a clearer picture of the dynamics of change in psychotherapy.

First, a contribution from Austria and Germany by Bachler et al. brings the results of an empirical study on 48 multi-problem families. They performed a home-based family therapy, consisting of one or two face-to-face sessions per week over an average of 28.8 months. Three years after the end of the treatment, the patients were significantly improved in terms of symptoms, ego strength and social skills. From Israel, Tzur Bitan et al. present the results of their empirical study on the dynamics of change within Crisis-Focused Psychotherapy. After the treatment the patients were significantly improved in terms of symptoms and perceived impotence (what the authors call “Entrapment”). From Sweden, Werbart et al. present a thematic analysis based on interviews with patients conducted at the end of their psychoanalytic treatment. They report clinically meaningful improvements in three main domains: Work and Achievements, Love and Relationships, the Self (awareness and agency). Del Giacco et al. from Spain and Italy, present an empirical study on stable patterns of verbal and non-verbal communication between patient and therapist. In particular, they analyze the contribution of those two aspects in the construction of the therapeutic alliance with depressed patients. Our own study, which involved colleagues from Italy, UK, Austria, Germany and Belgium, de Felice et al. analyzed how four good-outcome and four poor-outcome psychotherapies evolve over time, displaying an increase in flexibility at the end of the psychotherapies of the first group. The study showed not only how to quantitatively describe psychotherapy as a network, but also identified the main principles on which this evolution is based. From Spain, the colleagues Arias-Pujol and Anguera analyzed the changes in the therapist communication strategies during a group psychotherapy with six adolescents. Takagaki et al. present the results of an empirical study on 1,042 patients with depression in Japan, showing how the trait mindfulness is a protective factor against functional impairment and avoidance. Uniting researchers from Austria, Germany and Denmark, Schiepek et al. present an empirical comparison between seven different measures to identify phase transitions. This comparison, made on both simulated and real series, had a positive outcome showing convergent results and, thereby, allowing to reliably identify phase transitions in psychotherapy. Finally, a collaboration between experts in Italy, Germany and Austria by Gennaro et al. presents an analysis of 95 dreams occurring within a single psychoanalytic psychotherapy. Based on multiple correspondence analysis, the authors highlight the evolution of dreams in relation to the unconscious themes present within them.

We can now get back to the two main questions at the heart of this Research Topic.

Regarding the *processes that promote change in psychotherapy* we note the possibility of abstracting two essential ingredients from the presented contributions, regardless of the specific therapeutic approach. The first can be defined as *Awareness* (or Self-Reflection). It consists of making the patient aware of his/her dysfunctional relational models. The second, which we term *Restructuring Experience*, consists of offering the patient a restructuring relational experience. For example, the psychotherapist keeps on listening the patient even when he/she seems very rejecting or seems to do everything possible to break the relationship with him/her. Therefore, a Restructuring Experience can take place when we, as psychotherapists, are able to offer the patient a different and more functional way of being with us, in comparison with what the patient experienced in his/her previous relational models. The Awareness and Restructuring Experience can therefore be regarded as two essential ingredients of psychotherapy (“control parameters” according to the language used for dynamic systems) that provide the patient with the psychic energy necessary to move his/her mind from a stable and pathological relational model to a more functional one (process defined “order-to-order transition” in terms of dynamic systems).

Regarding the *precursors of change in psychotherapy* we identify two main groups of indices that are in accordance with the previous literature (e.g., Scheffer et al., 2009, 2018; Gorban et al., 2010, 2021; Gumz et al., 2010, 2012; Schiepek et al., 2014, 2017, 2020; Halfon et al., 2016, 2019; de Felice et al., 2019a,b; Olthof et al., 2019). The first group refers to those indices of *Coherence* of the psychotherapy process variables^1^. As shown in previous studies, a peak of coherence within process variables leads to a subsequent phase transition, or therapeutic restructuring (e.g., Haken and Schiepek, 2006; Scheffer et al., 2009, 2018; Gorban et al., 2010, 2021). This property has also been demonstrated in other scientific domains such as physiology, chemistry, economics. In psychotherapy, this phenomenon is observed in clinical contexts when the patient can acquire the awareness of his/her own dysfunctional relational models and is able to observe them across all important domains of his life (e.g., in the significant relations of the past, in the significant relations of the present, in work, and, sometimes, also in the sessions with the therapist). Hence, this is translated into an increase of coherence in the patient's narratives and personal history. In quantitative terms, previous studies assessed coherence in psychotherapy with the following indices, extracted from a given group (i.e., matrix) of process variables: (a) the sum of their Pearson coefficients in absolute value at a given time; (b) the percentage of variance explained by their first principal component at a given time; (c) their autocorrelation (i.e., correlation between the matrix of process variables at time *t* and the same matrix at time *t-1*). The second group of precursors of change refers to the indices of *Flexibility* of the psychotherapy process variables. Indeed, it has been shown in different studies how a peak in flexibility leads to a therapeutic restructuring (e.g., Schiepek et al., 2014, 2017, 2020; Halfon et al., 2016, 2019; de Felice et al., 2019b). Also this latter property is in agreement with results from other scientific domains outside psychology (e.g., chemistry, physics, economics). In psychotherapy, this phenomenon in clinical terms is observed when the patient, during the process of change in his/her pathological relational models, oscillates between past, more pathological, and new, more functional, but unfamiliar, organizations. Hence, this is translated into an increase in the flexibility of the patient's in-session narratives, and an easier access to elements of novelty with respect to his/her personal history. In quantitative terms, previous studies measured flexibility in psychotherapy with the following indices, extracted from a given group (i.e., matrix) of process variables: (a) the Shannon entropy applied to the distribution of their eigenvalues at a given time; (b) the Distribution X Fluctuation of one or more process variables (i.e., “Dynamic Complexity,” Haken and Schiepek, 2006).

We believe the present Research Topic contributes to the existing literature by shedding light on the dynamics and precursors of change in psychotherapy.

We again thank all the authors for their contribution to this Research Topic, and we sincerely wish all colleagues a pleasant reading.

## Author Contributions

All authors listed have made a substantial, direct and intellectual contribution to the work, and approved it for publication.

## Conflict of Interest

The authors declare that the research was conducted in the absence of any commercial or financial relationships that could be construed as a potential conflict of interest.

## Publisher's Note

All claims expressed in this article are solely those of the authors and do not necessarily represent those of their affiliated organizations, or those of the publisher, the editors and the reviewers. Any product that may be evaluated in this article, or claim that may be made by its manufacturer, is not guaranteed or endorsed by the publisher.

## Footnotes

^1^These indices can be regarded as a second-order measures derived from specific calculations performed on a specific group of process variables, regardless of whether they are physiological, relational, or linguistic variables (for details see de Felice et al., 2019b).
